# Artificial intelligence-based echocardiography assessment to detect pulmonary hypertension

**DOI:** 10.1183/23120541.00592-2024

**Published:** 2025-05-12

**Authors:** Mahan Salehi, Samer Alabed, Michael Sharkey, Ahmed Maiter, Krit Dwivedi, Tarik Yardibi, Mona Selej, Abdul Hameed, Athanasios Charalampopoulos, David G. Kiely, Andrew J. Swift

**Affiliations:** 1 Department of Infection, Immunity and Cardiovascular Disease, University of Sheffield, Sheffield, UK; 2 NIHR Biomedical Research Centre, Sheffield, UK; 3 Janssen Research & Development, LLC, Raritan, NJ, USA; 4 Sheffield Pulmonary Vascular Disease Unit, Royal Hallamshire Hospital, Sheffield, UK; 5 Joint senior authors; 6 at time of study

## Abstract

**Background:**

Tricuspid regurgitation jet velocity (TRJV) on echocardiography is used for screening patients with suspected pulmonary hypertension (PH). Artificial intelligence (AI) tools, such as the US2.AI, have been developed for automated evaluation of echocardiograms and can yield measurements that aid PH detection. This study evaluated the performance and utility of the US2.AI in a consecutive cohort of patients with suspected PH.

**Methods:**

1031 patients who had been investigated for suspected PH between 2009–2021 were retrospectively identified from the ASPIRE registry. All patients had undergone echocardiography and right heart catheterisation (RHC). Based on RHC results, 771 (75%) patients with a mean pulmonary arterial pressure >20 mmHg were classified as having a diagnosis of PH (as per the 2022 European guidelines). Echocardiograms were evaluated manually and by the US2.AI tool to yield TRJV measurements.

**Results:**

The AI tool demonstrated high interpretation yield, successfully measuring TRJV in 87% of echocardiograms. Manually and automatically derived TRJV values showed excellent agreement (intraclass correlation coefficient 0.94, 95% CI 0.94–0.95) with minimal bias (Bland–Altman analysis). Automated TRJV measurements showed equally high diagnostic accuracy for PH as manual measurements (area under the curve 0.88, 95% CI 0.84–0.90 *versus* 0.88, 95% CI 0.86–0.91).

**Conclusion:**

Automated TRJV measurements on echocardiography were similar to manual measurements, with similarly high and noninferior diagnostic accuracy for PH. These findings demonstrate that automated measurement of TRJV on echocardiography is feasible, accurate and reliable and support the implementation of AI-based approaches to echocardiogram evaluation and diagnostic imaging for PH.

## Shareable abstract

**The US2.AI, an artificial-based tool for evaluating echocardiograms, can accurately and reliably measure tricuspid regurgitation jet velocity in patients with pulmonary hypertension and could aid in the early detection of this disease** https://bit.ly/48OYBVe

## Graphical abstract

**Figure:**
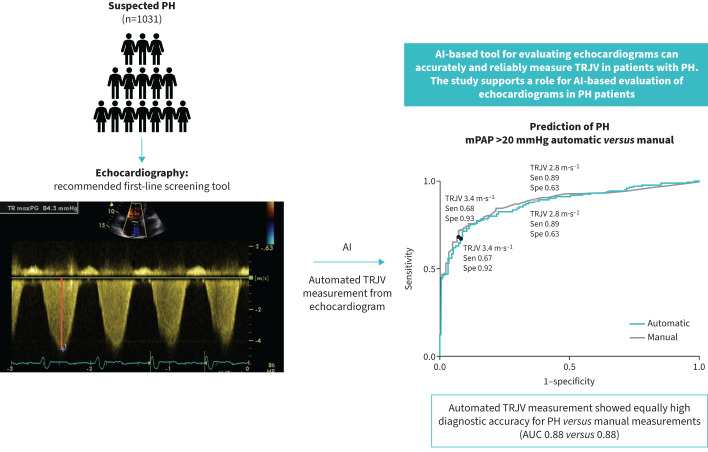
AI: artificial intelligence; AUC: area under the curve; mPAP: mean pulmonary arterial pressure; PH: pulmonary hypertension; Sen: sensitivity; Spe: specificity; TRJV: tricuspid regurgitation jet velocity.

## Introduction

Timely detection of pulmonary hypertension (PH) is challenging. Due to the nonspecific symptoms at presentation, such as exertional dyspnoea and fatigue, diagnostic delays are common, often in excess of 2 years [1–3]. PH has a number of underlying causes, including cardiorespiratory disorders, to which PH symptoms can be misattributed [4]. Earlier diagnosis of PH, together with an accurate identification of the underlying cause, is important for ensuring prompt initiation of appropriate treatment and better patient outcomes [5, 6].

Right heart catheterisation (RHC) is an invasive test that allows direct measurement of pulmonary artery pressures and remains the gold standard for diagnosing PH [7]. PH is defined as a mean pulmonary arterial pressure (mPAP) of >20 mmHg [8]; this threshold was recently reduced from an mPAP ≥25 mmHg [9]. For patients with suspected PH, an established, noninvasive first-line screening tool is transthoracic echocardiography [8]. As noted in the 2022 European Society of Cardiology (ESC) and European Respiratory Society (ERS) guidelines for the diagnosis and treatment of PH, a number of standard echocardiogram-derived parameters can be used to predict the presence of PH [8]. Tricuspid regurgitation jet velocity (TRJV) is considered the most reliable predictor, in which a TRJV >3.4 m·s^−1^ is associated with a high probability of PH and ≤2.8 m·s^−1^, a low probability [8]. In addition to TRJV, there are eight other echocardiographic parameters that can raise suspicion of PH, split into three categories: 1) the ventricles (right ventricle (RV)/left ventricle (LV) basal diameter or area ratio >1; flattening of the interventricular septum; tricuspid annular plane systolic excursion (TAPSE):systolic pulmonary artery pressure (sPAP) ratio <0.55 mm·mmHg^−1^); 2) the pulmonary artery (right ventricular outflow tract Doppler acceleration time (RVOT AT) <105 ms and/or mid-systolic notching; early diastolic pulmonary regurgitation velocity >2.2 m·s^−1^; pulmonary arterial diameter >aortic root diameter or 25 mm); and 3) the inferior vena cava (IVC) and right atrium (RA) (IVC diameter >21 mm with decreased inspiratory collapse; RA area >18 cm^2^) [8]. Echocardiography is noninvasive, widely available and can detect a range of cardiac disorders, contributing to its appeal. Moreover, besides its screening value, a number of echocardiographic appearances have also been shown to have prognostic value in patients with PH [10–12]. However, while it allows for accurate measurement of the pulmonary circulation, echocardiography remains operator-dependent and prone to error with moderate precision [13] and with a reasonably high level of interobserver variability between measurements [14].

To assist with echocardiographic diagnosis of PH, machine-learning tools have been developed [15–18]. The US2.AI, for example, is based on convolutional neural networks and automatically evaluates echocardiography images to provide anatomical and functional information about the heart, including TRJV, right atrial area, ventricular diameter and atrial pressure. The tool, which is commercially available and has received US Food and Drug Administration clearance, classifies cine loop images according to standard views before performing annotations to yield quantifiable metrics. The design, training and testing of the tool have been described in detail [19, 20]. In brief, the tool was trained using a total of 1145 echocardiograms from 1076 patients with heart failure and validated using an internal holdout approach on 406 echocardiograms from 406 patients. The tool was also tested in two external datasets with a total of 32 270 echocardiograms from 9910 patients [19, 20]. Although the US2.AI tool was developed to aid the diagnosis of heart failure, it does yield information pertinent to the diagnosis and characterisation of PH. This study aimed to further evaluate the performance and utility of automated TRJV measurement on echocardiography in a “real-world” consecutive cohort of patients with suspected PH based on their RHC-derived mPAP in a tertiary UK centre.

## Methods

### Study design and dataset

Patients who had been assessed for suspected PH at the Sheffield Pulmonary Vascular Disease Unit from 2009 and 2021 were identified from the ASPIRE (Assessing the Spectrum of Pulmonary Hypertension Identified at a Referral Centre) registry [21] for inclusion in this retrospective analysis**.** All included patients underwent both echocardiography and RHC as part of routine clinical care within a maximum timeframe of 6 months.

Echocardiography was performed at the Sheffield Teaching Hospitals NHS Foundation Trust by trained cardiac physiologists using Powervision 6000 and 8000 machines manufactured by Toshiba (Japan) or Vivid machines manufactured by General Electric (USA). RHC was performed by experienced PH consultants using standard techniques. Briefly, this involved using a balloon-tipped 7.5 French thermodilution catheter (Becton-Dickinson, Franklin Lakes, NJ, USA) introduced *via* a Swan-Ganz catheter, usually *via* the internal jugular vein. Full details of exclusion and inclusion criteria have been previously published [22].

Ethical approval was granted by the local ethics committee and institutional review board (ASPIRE, reference c06/Q2308/8; REC 17/YH/0016) and all patients provided written informed consent. All data were handled in accordance with local information governance policy.

### RHC diagnosis of PH

Based on RHC results, patients were classified as having or not having a diagnosis of PH or pre-capillary PH according to their documented mPAP values and established diagnostic criteria as defined by the 2022 ESC/ERS guidelines [8]. PH was defined as an mPAP >20 mmHg. PH was further subclassified as pre-capillary PH, defined as an mPAP >20 mmHg and pulmonary vascular resistance (PVR) >2 Wood Units (WU) [8].

Patient classification based on older diagnostic criteria was also performed to assess whether there were any differences in results depending on the definition of PH and pre-capillary PH used. The definition of PH based on the 2015 ESC/ERS guidelines was mPAP ≥25 mmHg [9] and the definition of pre-capillary PH based on the 6th World Symposium on Pulmonary Hypertension recommendations [23] was mPAP >20 mmHg and PVR >3 WU. The results from these analyses are presented in the supplementary material.

### Echocardiogram evaluation

**FIGURE 1:**
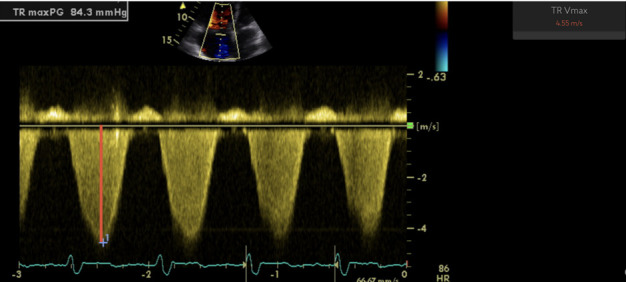
Echocardiographic measurement of peak tricuspid regurgitation jet velocity (TRJV). An echocardiogram showing a TRJV of 4.55 m·s^−1^ (red line), measured automatically, which corresponded to an estimated maximum tricuspid regurgitation pressure gradient of 84.3 mmHg, indicative of severe pulmonary hypertension (PH).

Echocardiograms were evaluated both manually and by the US2.AI. For all patients included in the study, the formal clinical reports of the echocardiograms were assessed and any documented manual measurements were recorded. The echocardiograms were also evaluated by the US2.AI software, which provided automated measurements, including TRJV, RV/LV ratio and RA area, among other measures. Failure of any individual measurement was recorded. Figure 1 illustrates the measurement of the TRJV. Right atrial pressure (RAP) was estimated based on the collapsibility of the IVC [24]. Systolic pulmonary artery pressure (sPAP) measurements were calculated from the manual and automated TRJV and RAP measurements, as follows: sPAP=(4×TRJV^2^)+RAP [25].

### Statistics

Statistical analysis and graph production were performed using RStudio (2022.07.1 running R 4.2.1) and Prism (version 9.4.1; San Diego, CA, USA). Continuous data were compared using the paired t-test and categorical data were compared using the chi-squared test. For paired tests, samples for which measurements were available from both AI and manual techniques were used. The significance threshold was set at p<0.05. No imputation of missing values was performed.

Agreement between automated and manual echocardiogram measurements was assessed using the intraclass correlation coefficient (ICC), with strength of the agreement based on the following established thresholds: ICC<0.2 (no agreement), ICC=0.2–0.4 (poor), ICC=0.4–0.6 (moderate), ICC=0.6–0.8 (good) and ICC>0.8 (excellent) [26]. Bias between the automated and manual measurements was also assessed by Bland–Altman analysis; these results are presented in accordance with published guidelines [27].

The accuracy of manual and automated TRJV measurements for the diagnosis of PH was assessed using receiver operating characteristic (ROC) analysis, using established TRJV thresholds of 2.8 m·s^−1^ and 3.4 m·s^−1^ for low and high probability, respectively [8]. Diagnostic accuracy was evaluated by obtaining the area under the curve (AUC). Accuracy, sensitivity and specificity were obtained for the aforementioned RHC-derived definitions of PH and pre-capillary PH. ROC results for manual and automated TRJV measurements were also compared using the “roc.test” function with DeLong's method [28].

For the estimated sPAP values derived from manual and automated echocardiogram evaluation, comparisons were made against RHC-derived sPAP values using linear regression models.

## Results

### Patients

**FIGURE 2:**
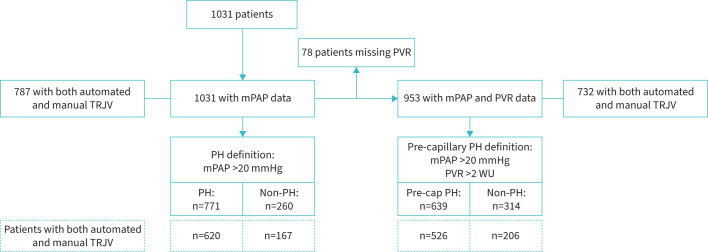
Flow chart of the study population and analysed subgroups based on the European Society of Cardiology/European Respiratory Society 2022 Guidelines. mPAP: mean pulmonary arterial pressure; PH: pulmonary hypertension; PVR: pulmonary vascular resistance; TRJV: tricuspid regurgitation jet velocity; WU: Wood Unit.

The study flow is provided in figure 2. In total, 1031 patients with suspected PH were included in the study (mean age 64±14 years, 66% female; supplementary table 1). mPAP was available in all cases. Of these patients, 771 had a PH diagnosis based on the 2022 ESC/ERS guideline definition (mPAP threshold of >20 mmHg) [8]. Diagnosis of pre-capillary PH was feasible in 953 patients (92.4%); missing cases (n=78) were due to noncoded pulmonary artery wedge pressure. Among the 953 patients, 639 patients were classified as having pre-capillary PH (mPAP >20 mmHg, PVR >2 WU) [8].

### Manual and automated TRJV measurements

Out of the 1031 cases, TRJV was manually read in 820 (80%) cases compared with 894 (87%) automated cases, with a large overlap of 787 cases (supplementary table 2), indicating AI overperforming manual readings in terms of interpretation yield (p<0.001, chi-squared 18.42); this was seen across both PH and non-PH cohorts.

**FIGURE 3:**
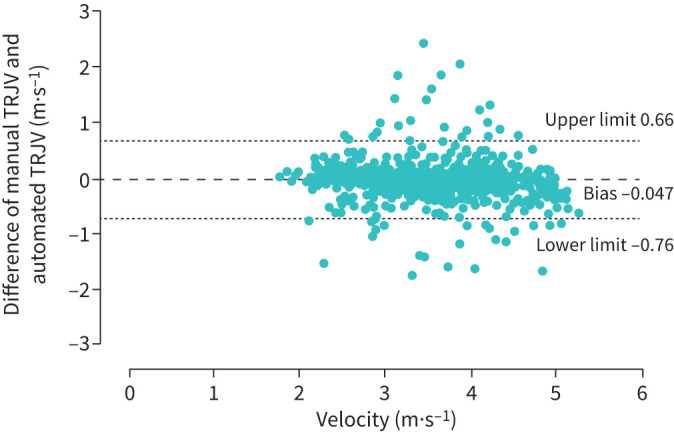
Bland–Altman plot of manual minus the automated tricuspid regurgitation jet velocity (TRJV) (m·s^−1^) measurement indicating excellent agreement and minor bias. Mean difference −0.05±0.36, 95% confidence interval −0.76–0.66. Slight scatter is likely due to variability and degree of error in both automated and manual measures.

There was no statistical difference between the manual TRJV measurements (3.85±0.71 m·s^−1^) and automated measurements (3.72±0.70 m·s^−1^) in patients with pre-capillary PH (p=0.439; table 1). This was also the case for non-pre-capillary PH patients (2.75±0.44 m·s^−1^ and 2.72±0.48 m·s^−1^; p=0.517). Manual and automated TRJV measurements demonstrated excellent agreement (ICC 0.94, 95% CI 0.94–0.95) and minimal bias on Bland–Altman analysis (mean difference −0.05±0.36, 95% CI −0.76–0.66; figure 3).

**TABLE 1:** Distribution of available echocardiography features for diagnosing pulmonary hypertension (PH) from all cases and for pre-capillary PH using 2022 European Society of Cardiology/European Respiratory Society diagnostic criteria (mPAP >20 mmHg and PVR >2 WU)

Measure	All cases (n=1031)			Diagnostic criteria: mPAP>20 mmHg, PVR>2 WU (n=953)					
				Pre-capillary PH (n=639)			Non-pre-capillary PH (n=314)		
	Automated	Manual	p-value	Automated	Manual	p-value	Automated	Manual	p-value
TRJV, m·s^−1^	3.45±0.80	3.56±0.82	0.238	3.72±0.70	3.85±0.71	0.439	2.72±0.48	2.75±0.44	0.517
RV/LV ratio	0.98±0.31	1.10±0.33	0.676	1.15±0.32^#^	1.17±0.31	0.426	0.87±0.21^¶^	0.87±0.19	0.891
Right atrial area	16.55±6.00	0	NA	17.40±6.10	0	NA	14.86±5.55	0	NA

Data are presented as mean±sd. LV: left ventricle; mPAP: mean pulmonary arterial pressure; PVR: pulmonary vascular resistance; RV: right ventricle; TRJV: tricuspid regurgitation jet velocity; WU: Wood Unit; NA: not available. ^#^: n=108; ^¶^: n=61. The p-values correspond to paired t-tests between the automated and manual measures.

Measurements of “other pulmonary hypertension echocardiographic signs”, including RV/LV ratio and RA were also collected automatically but analyses were only successful in 69% and 43% of cases, respectively. Manual measurements were also insufficient, thus limiting comparison analyses for these additional echocardiographic parameters.

### Manual and automated sPAP measurements

Although estimation of sPAP values was possible from all manual and automated TRJV values, in total, 754 sPAP values were analysed as, in a small number of cases (n=33), sPAP from RHC was unavailable.

Estimated sPAP derived manually (59.7±25.1 mmHg) was not significantly different to estimated values derived from automated TRJV (57.9±23.7 mmHg; p=0.17). sPAP measured directly on RHC showed excellent agreement with estimated sPAP derived from either manual (ICC 0.85, 95% CI 0.83–0.87) or automated (ICC 0.83, 95% CI 0.80–0.85) TRJV measurements from echocardiography, with minimal bias on Bland–Altman analysis (3.9±18 mmHg, 95% CI −32.0–39.0 for manual TRJV; 5.6±19 mmHg, 95% CI −31.0–42.0 for automated TRJV; supplementary figure 1). Agreement was also visualised using a linear regression model (r=16.9±1.6 for manual TRJV; r=17.2±1.7 for automated TRJV; supplementary figure 2).

### Diagnostic accuracy of TRJV for PH

The diagnostic accuracy of manual and automated TRJV measurements from echocardiography for PH was assessed according to the latest 2022 ESC/ERS guidelines criteria for PH and pre-capillary PH [8]. Little difference in diagnostic accuracy, sensitivity or specificity was demonstrated between manual and automated TRJV measurements, with similar AUC values for both TRJV thresholds of 2.80 m·s^−1^ and 3.4 m·s^−1^ (table 2). A 3.40 m·s^−1^ threshold, measured manually and automatically, provided accuracy of 73% and 72%, a sensitivity of 68% and 67%, and a specificity of 93% and 92%, respectively for diagnosing PH. Sensitivity increased with the lower TRJV threshold of 2.80 m·s^−1^ compared with 3.40 m·s^−1^ for both automated and manual measurements.

**TABLE 2:** Receiver operating characteristic analysis comparing the diagnostic accuracy of tricuspid regurgitation jet velocity (TRJV) for pulmonary hypertension (PH) and pre-capillary PH using automated and manual measurements

		Manual		Automated	
	TRJV threshold	PH (n=787)	Pre-capillary PH (n=732)	PH (n=787)	Pre-capillary PH (n=732)
		mPAP >20 mmHg	mPAP >20 mmHg, PVR >2 WU	mPAP >20 mmHg	mPAP >20 mmHg, PVR >2 WU
**AUC**		0.88 (0.86–0.91)	0.90 (0.88–0.92)	0.88 (0.84–0.90)	0.89 (0.86–0.91)
**Accuracy**	2.80 m·s^−1^	0.84 (0.81–0.96)	0.83 (0.81–0.86)	0.83 (0.81–0.86)	0.83 (0.80–0.85)
	3.40 m·s^−1^	0.73 (0.70–0.76)	0.78 (0.75–0.81)	0.72 (0.69–0.75)	0.76 (0.73–0.79)
**Sensitivity**	2.80 m·s^−1^	0.89 (0.87–0.92)	0.92 (0.90–0.95)	0.89 (0.86–0.91)	0.92 (0.90–0.94)
	3.40 m·s^−1^	0.68 (0.64–0.71)	0.72 (0.68–0.76)	0.67 (0.63–0.70)	0.70 (0.66–0.74)
**Specificity**	2.80 m·s^−1^	0.63 (0.56–0.69)	0.61 (0.54–0.67)	0.63 (0.56–0.70)	0.60 (0.52–0.67)
	3.40 m·s^−1^	0.93 (0.89–0.96)	0.93 (0.89–0.96)	0.92 (0.88–0.96)	0.90 (0.86–0.94)

Data are presented as mean (95% CI). AUC: area under the curve; mPAP: mean pulmonary arterial pressure; PVR: pulmonary vascular resistance; WU: Wood Unit. The diagnostic accuracy of manual and automated TRJV measurements from echocardiography for PH (mPAP >20 mmHg) and pre-capillary PH (mPAP >20 mmHg and PVR >2 WU) was assessed according to the 2022 European Society of Cardiology/European Respiratory Society guidelines [8]. Includes patients with both automated and manual TRJV values; 787 patients with mPAP data (PH, n=620; no PH, n=167); 732 patients with mPAP and PVR data (pre-capillary PH, n=526; non-pre-capillary PH, n=206).

**FIGURE 4:**
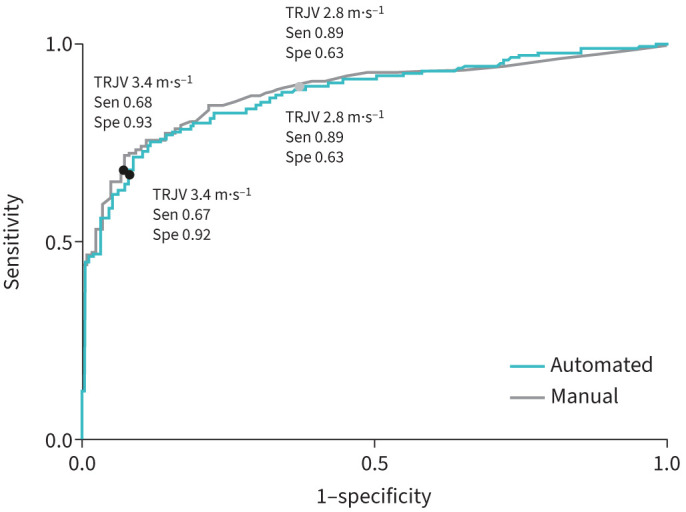
Receiver operating characteristic curve comparing automated (green) and manual (grey) measurements for diagnostic accuracy of tricuspid regurgitation jet velocity (TRJV) at low (black) and high (grey) thresholds for mean pulmonary arterial pressure ≥20 mmHg (p=0.31). Sen: sensitivity; Spec: specificity.

Comparing the ROC curves for PH and pre-capillary PH found no significant difference between manual and automated TRJV measurements (p=0.11–0.31; figure 4 and supplementary figure 3). The small differences in TRJV estimates from the AI and manual methods for the PH group do not impact the overall diagnostic accuracy (due to the margin between mean measurements and thresholds considered).

Results were similar using the older definitions of PH and pre-capillary PH (supplementary tables 3 and 4).

## Discussion

Echocardiography plays an important role in the early detection of PH, with peak TRJV acting as a key variable for assigning the echocardiographic probability of PH [8]. Automated evaluation of echocardiograms is appealing and can yield metrics that are of diagnostic and prognostic value in PH. This study evaluated the performance of the US2.AI, an existing commercial machine-learning tool, on echocardiograms from a retrospective cohort of 1031 patients undergoing investigation for suspected PH. Patients with PH were retrospectively identified from the cohort on the basis of RHC measurements and established diagnostic criteria. TRJV measurements derived manually and automatically from the echocardiograms showed strong agreement and high diagnostic accuracy for PH; this was observed using the more recently redefined diagnostic criteria for PH (as per 2022 ESC/ERS guidelines) [8] and also with the previous definition (as per 2015 ESC/ERS guidelines) [9].

To the best of our knowledge, this is the first study to date to evaluate the use of an automated measurement of TRJV on echocardiography in a population of patients with suspected PH. Automated measurements of TRJV were taken in 87% of all cases, demonstrating a high interpretation yield, and correlated highly with invasive RHC measurements. There was also strong agreement, minimal bias and similar diagnostic performance between automated and manually derived measurements. Overall, the diagnostic accuracy of echocardiographic TRJV measurements is in good agreement with reported literature [11, 29–31]. Our results demonstrate that an automated approach to TRJV measurement is accurate, reliable and robust in detecting suspected PH, supporting its clinical use. Automation of echocardiographic TRJV measurements also provides additional efficiencies in terms of workflow and time to measurements. For example, automated TRJV measurement in regular clinical practice could facilitate the consideration of potential PH and reduce time to diagnosis. We do however acknowledge that while the PH definition has been updated in the 2022 ESC/ERS guidelines [8], with lowering of the mPAP threshold, echocardiography parameter thresholds such as TRJV have remained the same. This may, especially for those with a mild elevation of mPAP, have the potential for underdiagnosis of PH. As expected, ROC analysis showed that the lower TRJV threshold of 2.80 m·s^−1^ was more sensitive (89–92%) than the higher threshold of 3.40 m·s^−1^ (67–72%) for PH and pre-capillary PH, for both automated and manual measurements. Although previous studies have produced mixed sensitivities, ranging from 60–100%, these tools have been trained and tested in a patient population with PH [15, 17, 32].

There are limitations to this study. First, the US2.AI tool was trained on patients with heart failure. Further training using a larger cohort of PH patients is likely to improve the generalisability of this tool. Second, we utilised a retrospective cohort identified from a registry and data were not systematically collected on other echocardiographic criteria and consequently, we could not evaluate performance for all of the ESC/ERS metrics. There is selection bias as the study cohort consisted of patients with suspected PH who had undergone both RHC and echocardiography. Consequently, the prevalence of PH in the cohort is high. Conducting a prospective study to assess all of the recognised PH metrics and to ensure that the AI is trained on a heterogenous cohort of patients with and without suspected PH will be an important future step before an AI tool can be implemented in routine evaluation of echocardiograms for PH patients.

In summary, we have demonstrated that an AI-based tool for evaluating echocardiograms can accurately and reliably measure TRJV in patients with PH. Automated TRJV measurements showed excellent agreement with manual measurements and were found to have high diagnostic accuracy for PH. The study supports a role for AI-based evaluation of echocardiograms in PH patients. Further studies are required to evaluate the diagnostic utility and automated measurement of other PH metrics on echocardiography, ideally in a prospective setting.

## Data Availability

The data underlying this article will be shared on reasonable request to the corresponding author.
